# Multiple asymptomatic papules following breast cancer treatment

**DOI:** 10.1002/ccr3.6447

**Published:** 2022-10-19

**Authors:** Kouki Chaima, Sellami Khadija, Bahloul Emna, Mnif Emna, Graja Soumaya, Kammoun Nadine, Gouiaa Naourez, Boudawara Tahya, Turki Hamida

**Affiliations:** ^1^ Department of Dermatology Hospital of Hedi Chaker Sfax Tunisia; ^2^ Anatomopathology Department Hospital of Hedi Chaker Sfax Tunisia

**Keywords:** acquired lymphangiectasia, breast cancer, dermoscopy, radiation therapy, warts

## Abstract

Acquired lymphangiectasia (AL) represents superficial lymphatic dilatation caused by different processes. It is a consequence of lymphatic damage by an external cause; leading to obstruction of local lymphatic drainage.1 We report a case of AL of the breast in a 45‐year‐old woman mimicking warts.

## Graphical abstract

Acquired lymphangiectasia (AL) represents superficial lymphatic dilatation caused by different processes. It is a consequence of lymphatic damage by an external cause, leading to obstruction of local lymphatic drainage. Radiotherapy remains of the major cause of lymphatic vessel injury. We report a case of AL of the breast in a 45‐year‐old woman treated for breast cancer mimicking warts.

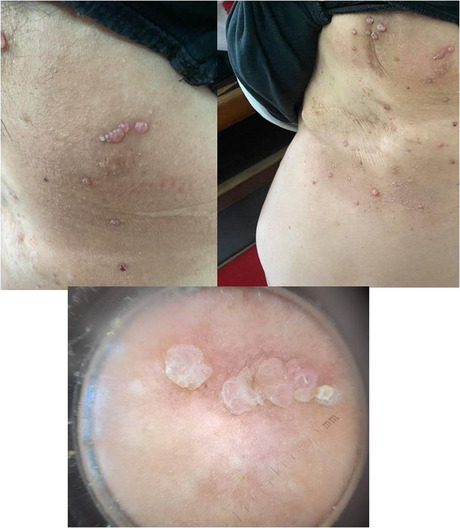

## 1 INTRODUCTION

Acquired lymphangiectasia (AL) represents superficial lymphatic dilatation caused by different processes. The damage can primarily be due to surgical intervention alone, irradiation alone, or by surgery and irradiation combined or secondary to scarring. It was first described as a complication of radical mastectomy.^1^ This condition most commonly occurs in adults. Clinical presentation of AL is dependent on the anatomical site and degree of lymphatic disruption. Typically, it manifests as translucent vesicles and papule associated to lymph edema.^2^ Different papers reported clinical mimics of AL including bullous lichen sclerosis et atrophicus, genital warts and infection with herpes simplex virus.^3^

In this report, we describe a case of cutaneous AL of the breast in a 45‐year‐old woman mimicking warts.

A 45‐year‐old woman presented to our dermatology department for multiple lesions on the left breast. She had a medical history of bilateral infiltrating duct cell carcinoma of the breast treated with a radical mastectomy, chemotherapy followed by Radiation Therapy (RT). She noticed multiple papules developing 2 years after surgery and RT. She was on remission since 1 year with homontherapy and under continuous follow‐up.

**FIGURE 1:**
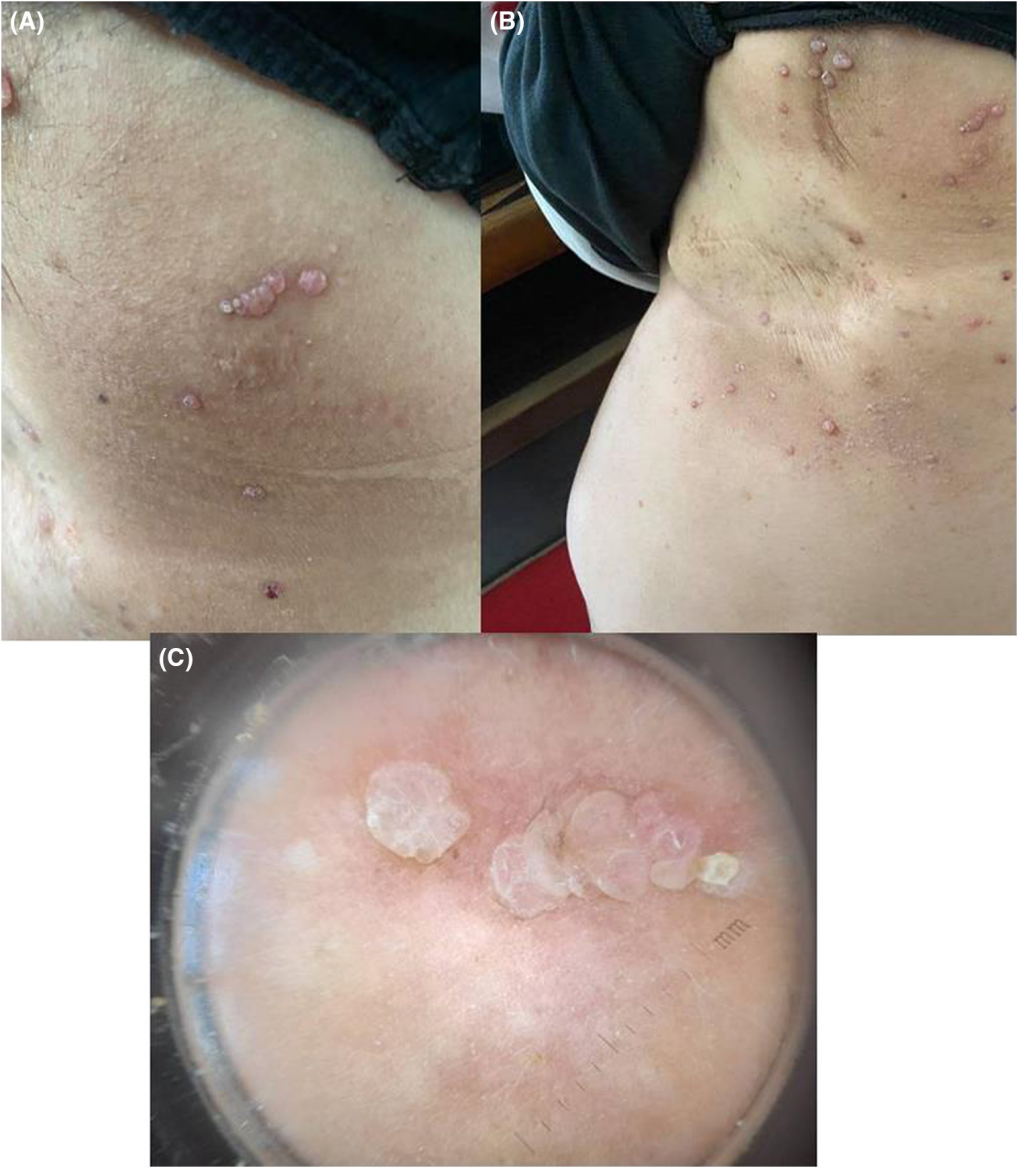
(A, B) Multiple grouped pinkish‐red translucent papules on the left anterior and lateral wall of the chest and on the left axilla region. (C) Dermoscopic image: multiple erythematous lacunae separated by white septa and linear vascular structures.

Cutaneous examination revealed multiple grouped pinkish‐red papules spreading over the left anterior, the lateral wall of the chest and on the left axilla region, some of which had a hyperkeratotic verrucous surface (Figure 1A,B). There was no evidence of lymphedema. Multiple erythematous lacunae separated by white septa and linear vascular structures were noticed on the polarized light dermoscopy (Figure 1C).

**FIGURE 2:**
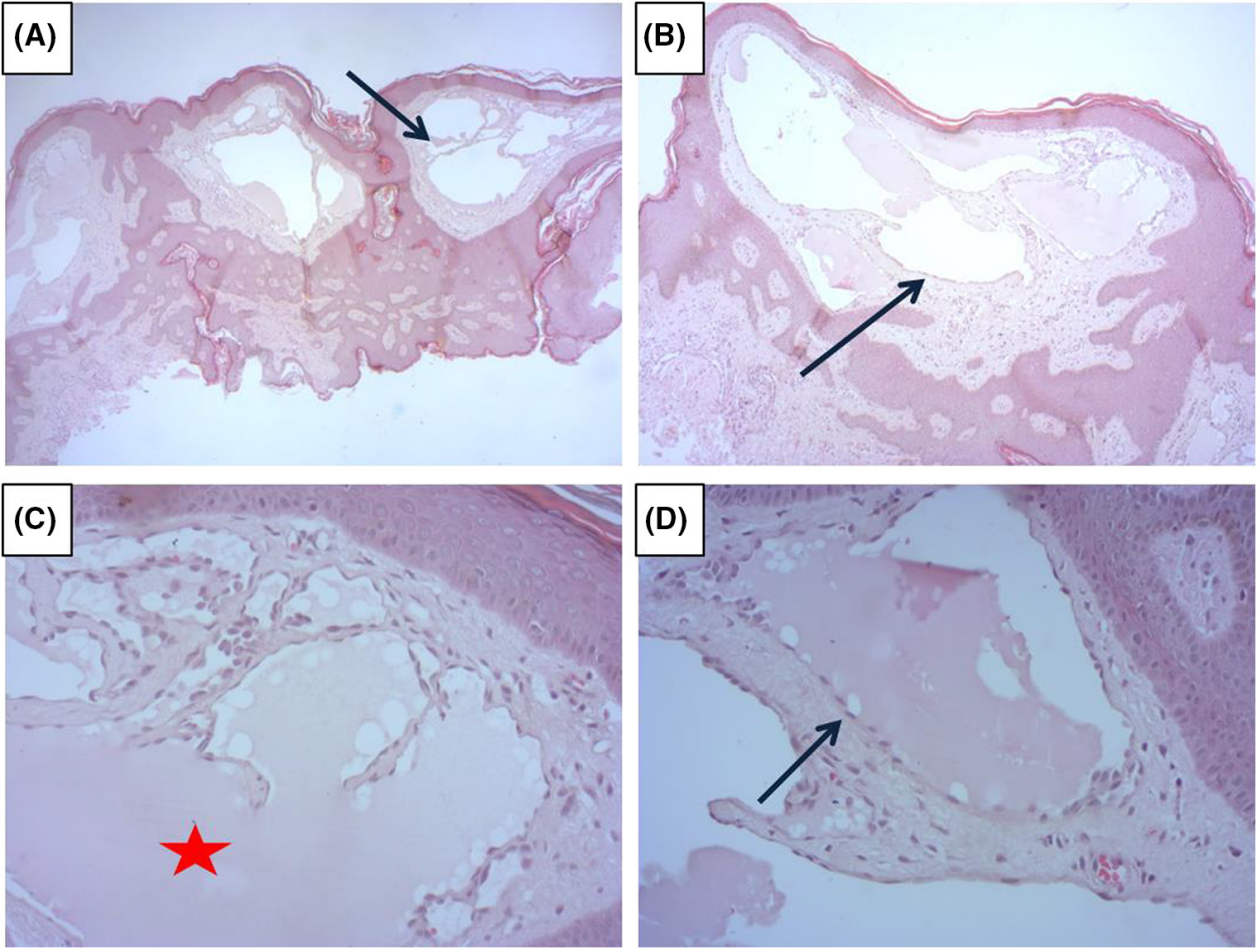
(A) Regular ortho‐keratotic epidermis, the dermis containing numerous vascular structures of the lymphatic type (HE*25). (B) Ecstatic lymphatic structures sometimes anastomotic (HE*50). (C) Lymphatic structures containing a chylous material (asterix) (HE*200). (D) Regular endothelium lining the lymphatic structure (HE*200).

Serial biopsies were taken from the prominent lesions. Histological examination showed numerous dilated lymphatic vessels in the superficial and papillary dermis lined by flattened endothelial cells, with mild hyperkeratosis. (Figure 2).

## 2 WHAT'S YOUR DIAGNOSIS?

Based on these clinical and histological findings, the diagnosis of AL was established. Finally, the treatment possibilities were explained to the patient including cryotherapy and laser.

Given the probability of recurrence, the patient refused any procedure. There was no extension of the lesion after 6 months of follow‐up.

Acquired lymphangiectasia represents acquired vesicular dilation of lymphatic channels caused by an external cause. In 1956, AL was first described as a complication of radical mastectomy.^1^ Due to the increase in surgical procedure, RT for different malignancies of breast and cervix; we are facing to an increased frequency of this entity in the past 20 years. Its mechanism remains unknown. AL is considered to be a consequence of lymphatic damage; leading to the obstruction of local lymphatic drainage.^1^ The fibrosis and lymphatic obstruction at the base of the reticular dermis and accumulation of lymph fluid in the dermal lymphatics with resultant increased pressure. This can lead to dilation of the superficial lymphatic channels with subsequent vesicle formation.^4^ RT itself is known to cause lymphangiectasia by causing lymphangiogenesis in the exposed area during the 1st year of therapy. Recently, it was found that there were an increased number of podoplanin‐positive lymphatic vessels and CD68‐positive histiocytes in cancer patients treated with the radiotherapy.^4^

Previously, Chiyomaru et al. notified external genitalia as the most frequent site. Furthermore, they concluded that the combination of surgery and RT (77%) was the most frequent preceding therapy, followed by surgery alone (18%) and irradiation alone (5%). The mean interval after RT to the onset of the AL was shorter after combination therapy (5.8 years) than after surgery (12.2 years) or irradiation (11.8 years) alone.^5^ Different delay was reported after the surgery varying from 3 to 26 years.^4^ The index case developed AL 2 years after surgery and radiotherapy, which is relatively short, compared with the other studies in the literature. It has also been associated with metastatic lymph node invasion.

Acquired lymphangiectasia is indistinguishable both clinically and histopathologically from lymphangioma circumscriptum (LC).^6^ The clinical findings of AL consist of clusters of translucent vesicles and papules 2–10 mm in size. Their colors range from colorless to purple, due to a varying content of red cells, which arise from minor hemangiolymphatic connections.5 Otherwise, AL can be difficult to identify clinically and evolve over time. New lesions appear vesicular and, as a result of tissue organization, they gradually become papular, as seen in our second case. Lesions typically occur on a background of existing lymphoedema.^3^, ^5^, ^6^ Occasionally, they may become pedunculated lesions, hyperpigmented macules and lesions with a verrucous surface that may be mistaken for viral warts.^7^ Similarly, in our patient, the lesions had a verrucous and hyperkeratotic appearance and the diagnosis of warts was suspected at first. This unusual presentation resembling warts has been reported previously on the vulva.^7^ One case of verruca vulgaris restricted to skin traumatized by RT was reported.^8^ In atypical cases, biopsy may be required. Coexisting lymphedema is a usual association in most cases of AL.^4^, ^6^ However, there was no associated lymphedema in our case. Our patient developed AL 2 years after surgery and RT, which is relatively short, compared with other studies in the literature. In fact, the absence of lymphedema is known to occur in AL following scarring in scrofuloderma, scleroderma, and cirrhosis. Similarly to our patient, Angoori Gnaneshwar Rao et al.^4^ notified that the occurrence of AL without lymphedema without an underlying disease is novel and interesting.

Most ACL lesions are asymptomatic but the course is chronic. Chronic drainage and oozing of lymph after trauma may provide a portal of entry for infection, giving rise to recurrent episodes of cellulitis.^4^, ^6^

Histologically, it reveals ectatic lymphatic spaces in the papillary dermis, which are lined by normal or flattened endothelial cells. The overlying epidermis may show hyperkeratosis and acanthosis. Although, there are no histological criteria to differentiate LC from AL some believe that the subcutaneous muscle‐coated cisternae characteristic of LC is absent in AL.^3^, ^4^ Nowadays, dermoscopy is a useful tool to the diagnosis. The most common findings are the lacunae which were found in our case and the hypopyon sign.^9^ Anna Elisa Verzì et al.^10^ described the dermoscopic of ALs after surgery and lymph node dissection for breast cancer. The white‐yellowish lacunae were surrounded by pale septa seen at dermoscopy. Moreover, the presence of reddish areas/red lacunae observed at dermoscopy in some lesions reflects the inclusion of blood cells within lymphatic vessels.^9^, ^10^, ^11^ The dermoscopic aspects seen in our patient are similar to those previously described.

Treating AL could be challenging. Recurrences are commonly detected with different rates. Management is centered on reducing the pressure in the lymph vessels. Physiotherapy, manual drainage, compressive bandages, and garments may be of some benefit.^5^, ^6^

Different treatment modalities: cryosurgery, electrocautery, sclerotherapy, and surgical excision have been utilized, with varying success. Antibiotic therapy may be necessary to treat infection and can also be given prophylactically to avoid recurrent episodes of erysipelas or cellulitis.^5^

Acquired lymphangiectasia has a good prognosis with no case of malignant transformation reported. However, long‐term follow‐up is needed.

## AUTHOR CONTRIBUTIONS

Dr Kouki Chaima is the guarantor of the content of the manuscript, included the data and analysis. Dr Sellami Khadija contributed to interpretation of data and revision of the manuscript. Dr Bahloul Emna contributed to data collection. Dr Kammoun Nadine and Mnif Emna contributed to analysis and interpretation of data, revised it critically. Dr Soumaya Graja and Guiaa Nawres provided the anatomopatological photo and contributed to its legend. Dr Hamida Tuki and Boudawara Tahya contributed to final approval of the version of the manuscript to be submitted.

## CONFLICT OF INTEREST

None.

## CONSENT

Written informed consent was obtained from the patient to publish this report in accordance with the journal's patient consent policy.
